# Four generations of SDHB-related disease: complexities in management

**DOI:** 10.1007/s10689-016-9946-9

**Published:** 2016-11-28

**Authors:** U. Srirangalingam, M. LeCain, N. Tufton, S. A. Akker, W. M. Drake, K. Metcalfe

**Affiliations:** Department of Endocrinology, St. Bartholomew’s Hospital, Queen Mary University of London, West Smithfield, London, EC1A 7BE UK

**Keywords:** Paraganglioma, Pheochromocytoma, Catecholamine, PGL4, SDHB mutation, Exon 4

## Abstract

SDHB mutations are linked to the familial paraganglioma syndrome type 4 (PGL4), which is associated with predominantly extra-adrenal disease and has high metastatic rates. Despite the lower penetrance rates in carriers of *SDHB* mutations compared to mutations in other paraganglioma susceptibility genes, the aggressive behavior of SDHB-linked disease warrants intensive surveillance to identify and resect tumors early. Patients with similar *SDHB* genotypes in whom the PGL syndrome manifests often exhibit very heterogeneous phenotypes. Tumors can arise in various locations, and management can be considerably different, depending on tumor site and pathology. We present a case series of five SDHB mutation carriers over four generations from the same family to illustrate the complexities in management.

## Introduction

Paragangliomas (PGLs) are highly vascular tumors arising in neural crest cells derived from neuroendocrine tissues associated with the autonomic nervous system and occur anywhere between the base of the skull and the pelvis [1]. Tumors originating from chromaffin cells of the adrenal medulla are termed pheochromocytomas. Both germline and somatic mutations in susceptibility genes, of which more than a dozen have been identified so far, drive disease, with familial forms of PGL accounting for at least 40% of all tumors [2]. The paraganglioma/pheochromocytoma susceptibility genes include the nuclear genes *SDHA*, *SDHB*, *SDH*C and *SDHD*, which encode the four subunits of the mitochondrial enzyme succinate dehydrogenase, involved in Krebs cycle oxidative phosphorylation and electron transport in the respiratory chain within mitochondria. The *SDHB* gene acts as tumor suppressor. Tumorigenesis is initiated with the ‘second’ hit and loss of heterozygosity at the SDH subunit gene locus, which leads to deactivation of the enzyme and impaired mitochondrial function [1]. Accumulation of the SDH substrate succinate leads to the stabilization of hypoxia-inducible factor α (HIFα), precipitating a state of mitochondrial ‘pseudohypoxia’ with consequent initiation of angiogenesis, proliferation and cell survival [2]. More recently, the role of the *SDHB* gene as a driver of hypermethylation and thus silencing of key genes involved in neuroendocrine differentiation have been proposed [3].

Mutations of *SDHB* gene predispose to the familial PGL syndrome type 4 (PGL4), characterized by a high incidence of extra-adrenal PGLs and a high rate of metastasis [4]. SDHB mutations carriers are predisposed to developing other tumors including renal cell carcinoma, gastrointestinal tumors (GIST) and rarely pituitary adenomas [5]. Despite incomplete penetrance, morbidity and mortality are considerably higher in disease associated with *SDHB* mutations than disease linked to other SDHx mutations, which generally follow a more benign course [5].

Here we report on a unique family with four generations affected by a *SDHB* mutation and highlight the heterogeneity of presentation and consequent variation in management strategies tailored to the individual carrier. All subjects or their guardians provided informed consent for this study. The familial mutation was previously reported in a study of UK subjects [4]. A summary of disease characteristics is provided in Table 1.

**Table 1:** SDHB mutation carriers—disease characteristics

	Subject 1 (proband)	Subject 2	Subject 3	Subject 4	Subject 5 (historical case)
Age at first surveillance	41	81	15	6	42
Disease characteristics	PGL head and neck PGL abdomen	PGL abdomen (benign)	PGL abdomen (locally invasive)	Asymptomatic carrier	PGL abdomen (distant metastasis) († aged 46)
Biochemistry at first surveillance	Abnormal	Abnormal	Abnormal	Normal	Not available
24 h urine NAdr	**+**	+	**+**	**–**	
24 h urine Adr	–	–	–	–	
24 h urine Dop	–	–	**+**	**–**	
Chromogranin A	+	Not available	–	Not available	
Chromogranin B	–	Not available	–	Not available	
BP at presentation (mm/Hg)	154/95	187/110	125/68	98/59	Not available
Treatment	Head and neck PGL − surgical excision + Gamma knife radiotherapy PGL abdomen − managed conservatively and under observation	Ongoing surveillance of abdominal PGL BP and diabetes control	Surgical excision	–	Radiotherapy
Current status	Stable disease	Stable disease	Asymptomatic, no evidence of recurrence	Asymptomatic, no evidence of disease	Deceased

### Case series

#### Subject 1 (index case)

**Fig. 1:**
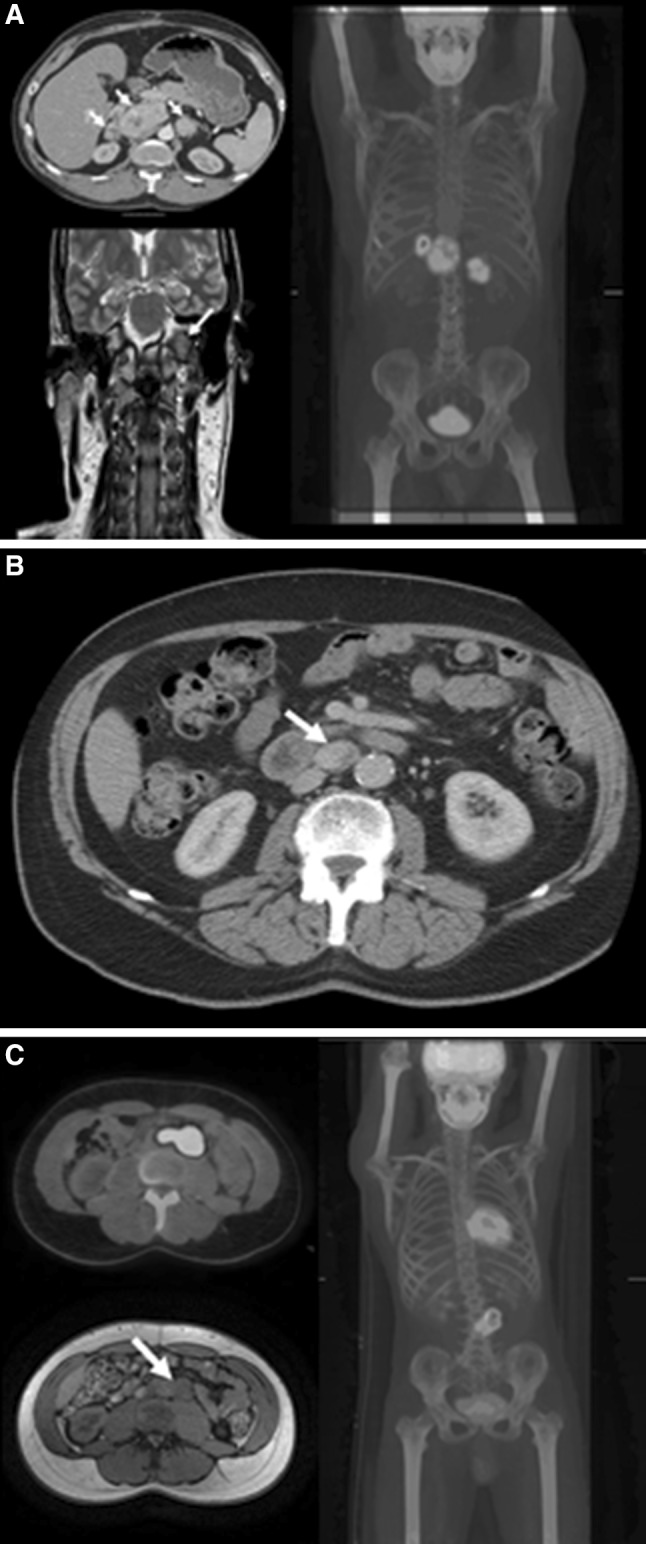
Imaging from subject 1 demonstrating abdominal and glomus vagale PGL; subject 2 with an abdominal PGL and subject 3 with a metastatic PGL involving the aorta

The index case presented at the age of 41 years with a history of dysphonia, dysphagia and palpitations. Examination revealed a left vocal cord palsy and a fasciculating tongue deviating to the left. Imaging identified a mass at the base of skull involving the left vagus and hypoglossal nerves, with erosion of the petrous bones into the internal auditory canal. Staging imaging identified additional bilateral abdominal lesions adjacent to the aorta and inferior vena cava (IVC). The left-sided lesion bordered the adrenal gland, and the right-sided lesion was intimately related to the celiac axis, displacing both the IVC and the portal vein (see Fig. 1a). A diagnostic mini-laparotomy by the initial investigating team resulted in extensive intraoperative bleeding and was inconclusive. Subsequent urinary noradrenaline level was found to be elevated at 787 nmol/24 h (normal range 100–560 nmol/24 h) with normal urine adrenaline. Chromogranin A was elevated at 111 nmol/L (normal range 0–60 nmol/L). MIBG scintigraphy was weakly positive in the abdomen, with no evidence of metastasis. A diagnosis of multiple PGLs was made. The invasive left jugular foramen tumor was debulked under adrenergic blockade, followed by gamma knife radiosurgery to the residual tumor. A bioplastic injection into the left vocal cord led to a near-normal voice. Surgical excision of the abdominal PGLs was deemed high risk on the basis of their proximity to the great vessels. The abdominal PGLs have thus been managed expectantly. The subject remains normotensive on adrenergic blockade with stable abdominal disease over 6 years. Genetic testing confirmed a novel missense variant of a G to A base substitution at nucleotide position 338 (c338G>A) in exon 4 of the *SDHB* gene. The mutation results in the substitution of the amino acid cysteine for the amino acid tyrosine at codon 113 (p.Cys113Tyr) [4].

#### Subject 2

The father of the index subject tested positive for the familial *SDHB* mutation following familial screening. He had a 20-year history of well-controlled hypertension and an 8-year history of type II diabetes mellitus. Screening CT imaging at the age of 81 years revealed a 2.8 cm retroduodenal mass, most likely a PGL (see Fig. 1b). Five years of radiological follow-up have shown a stable lesion, and urinary catecholamines have revealed an intermittently raised noradrenaline level. Given the patient’s age and the fact that he was normotensive on alpha blockade without progressive disease, it was decided to manage the disease conservatively.

#### Subject 3

The son of the index case was found to have elevated urine and plasma catecholamines on initial surveillance at the age of 15 years following his father’s diagnosis. He was normotensive (BP 125/68 mmHg), urinary noradrenaline was elevated at 2863 nmol/24 h (normal range 100–560 nmol/24 h), and the plasma normetanephrine was elevated at 3575 pmol/L (normal range <1180 pmol/L). Adrenaline and metanephrine levels were normal. An MRI scan revealed a single 5.8-cm retroperitoneal left paraaortic mass in the region of the duodenum, extending from just below the renal vessels to the level of aortic bifurcation (see Fig. 1c). This mass demonstrated avid uptake on FDG–PET scan, suggestive of a PGL. He was alpha blocked and consequently underwent surgery, where the tumor was found to be invading the aorta bifurcation. Furthermore, a lymph node inferior to the tumor was found to be involved intraoperatively. Aortic resection and insertion of a Dacron graft were required to fully remove the PGL. He made a good recovery. Over 4-year follow-up, the urinary metanephrines have been normal with no evidence of residual disease on radiological and functional imaging. He has been referred for vascular follow-up with respect to his aortic graft.

#### Subject 4

The younger son of the index subject tested positive for the familial *SDHB* mutation aged 6 years. He has remained symptom-free over a 3-year period with no evidence of PGL on surveillance.

#### Subject 5

It became clear that disease was present further back in the family history. The index subject’s grandfather presented in 1940 at the age of 42 years with abdominal pain on exertion. Laparotomy for suspected appendicitis was performed, where an ‘inoperable tumor wrapped around the aorta’ was identified. He was consequently treated with radiotherapy. He re-presented in 1943 complaining of back pain and was diagnosed with metastatic disease to the spine, for which he received spinal radiotherapy. He died at the age of 46. Though unconfirmed, the likely diagnosis was metastatic PGL.

## Discussion

We have presented a family of five subjects across four generations all affected by PGLs due to a familial missense *SDHB* mutation. Inactivating mutations of the *SDHB* gene are linked to the familial PGL syndrome type 4 [2]. Germline *SDHB* mutations account for an estimated 22–38% of hereditary pheochromocytomas/PGLs [6, 7]. They are inherited in an autosomal dominant manner without genomic imprinting, in contrast to other familial mutations in susceptibility genes, such as *SDHD*, where imprinting has been described, and disease is almost exclusively passed on by the father [8]. The estimated mean age of onset of disease for hereditary SDHB-related pheochromocytoma/PGLs is 34 years [9], but documented cases of malignant PGLs have occurred in childhood before the age of 10 years [9]. Compared to other familial paraganglioma syndromes, SDHB-related disease is characterized by low overall penetrance, a more aggressive phenotype and significant heterogeneity in presentation [4]. After correction for ascertainment, the lifetime penetrance of SDHB-associated disease has been estimated to be approximately 30% [10], compared to 75% disease penetrance by age 40 in carriers of SDHD mutations [5].

The family described here demonstrates the marked heterogeneity of disease associated with SDHB mutations. Despite the same mutation, we note disease presenting from childhood to old age, an aggressive disease phenotype in some individuals with local invasive disease and multiple tumors (subject 1), metastatic disease (subject 3) and seemingly benign PGL in subject 2. This diversity is reflected in the individualized management plans ranging from aggressive tumor debulking to leaving tumors in situ (subjects 1 and 2). Given the overall low penetrance of SDHB-related disease, we note that PGLs have presented in each generation of this family. This may suggest a mutation-specific preponderance for disease; however, a lack of clear genotype–phenotype correlation in SDHB mutations has previously been reported [4].

The diversity in disease characteristics and time of presentation within this family reiterates the importance of tailored management for each subject. SDHB mutation carriers require lifelong biochemical and clinical surveillance due to these variations in presentation and the considerable risk of metastatic disease. Such patients should be managed by a multidisciplinary team in a tertiary care setting. In our institution, a familial PGL clinic runs on a monthly basis in which families may be seen together and which is supported by the multidisciplinary team including a clinical nurse specialist, both adult and pediatric endocrinologists, a radiologist, an endocrine surgeon and a patient support group. Our current surveillance regimen includes annual plasma/urinary metanephrines, annual MRI of the abdomen and 2-yearly imaging of the neck, thoracic and pelvis. Surveillance starts from the age of 5 years and may be tailored to the individual child as tolerated. Intensive surveillance allows disease to be identified promptly, so that surgery can be instigated early to affect the natural history of disease (subject 3).

Subjects with SDHB-associated disease present a specific set of management dilemmas to the clinician—that of lowly penetrant disease, but with high metastatic potential and limited treatment options. Surveillance programs must allow disease to be identified early while not over-medicalizing and generating anxiety in carriers who are unlikely to ever develop disease.
